# QTL tools applied to forest breeding

**DOI:** 10.1186/1753-6561-5-S7-P47

**Published:** 2011-09-13

**Authors:** Luciano Medina-Macedo, Alexandre Coelho

**Affiliations:** 1 Biotechnological Processes, Federal University of Parana, Curitiba, Parana, 81531-990, Brazil; 2 Department of Agronomy, Federal University of Goias, Goiania, Goias, 74001-970, Brazil

## Background

In last decades, the progress of molecular techniques, bioinformatics tools and genome analysis equipment allowed genomes to be sequenced and analyzed for several plants. For Eucalyptus, the combination of quantitative trait loci (QTL) mapping with genomic analyses, has allowed the identification and validation of a few genomic regions involved in growth and wood quality characters. However, several forest companies still have difficulties to apply Marker Assisted Selection (MAS) at the operational level in their genetic improvement programs. Using as case study of a Eucalyptus hybrid progeny, which had QTLs mapped for growth and wood quality characters, the present study compared the possible outcomes of traditional selection used in forest breeding programs, where individuals are classified by genetic values (eBLUP), with selection based only on superior QTL genotypes (MAS).

## Methods

Genotype selection included 30 trees as the maximum number selected by both selection methods, an amount that corresponds to a 15% selection intensity in relation to the mapping population size used in this study. About 200 individuals of an interespecific hybrid progeny had 3 growth characters evaluated in the field and 5 wood quality characters predicted by Near Infra-Red Spectroscopy (NIRS). This progeny was genotyped with 76 microsatellite markers (SSR). An integrated genetic map was built at LOD 3 and 0.40 recombination fraction, composed by 65 SSR markers distributed in 12 linkage groups with total length of 1,365 cM and average distance among markers of 21 cM [1,2]. The genetic map built covered approximately 83% of the Eucalyptus genome estimated by Brondani et al. [3]. Due to the low density and large distance among markers obtained, QTL mapping was only carried out by single marker analysis using R, at p-values smaller than 0.01.

## Results

Out of the 65 mapped loci, 10 of them (~15%) showed association with a total of 12 QTLs; 10 loci were associated with only one character and 2 loci with two different growth characters. Three characters showed association to only one QTL. The average volume growth of the 30 individuals selected by MAS was 55% lower than the average of the 30 best individuals selected by phenotypic eBLUP values. One QTL was found for syringil/guayacil lignin ratio and the average of the 30 selected trees by MAS was 17% lower than the average of the best individuals selected by eBLUP. Two QTLs were found for wood density, diameter and lignin content. For density and lignin content, multiple selection for superior alleles at two QTL didn't restrict the number of selected trees, and the average values of individuals selected by MAS were respectively 45% and 83% lower than the average of the best individuals selected by eBLUP. The simultaneous selection for superior alleles at two QTL for diameter restricted from 30 to 23 the number of selected trees. The simultaneous selection for these two QTLs reduced the average value of the 23 individuals selected by MAS to almost half of that of the 23 best individuals selected by eBLUP. For cellulose yield, the simultaneous selection for superior alleles at three QTL reduced the number of selected trees from 30 to 29. The average value of MAS selected trees was 78% lower than the one of the 29 trees selected by eBLUP. In conclusion, for all traits evaluated, selection based on eBLUP was significantly more efficient than MAS. This result was expected, due firstly to the low marker density of our experiment and hence low power to detect all QTLs involved in the trait. Secondly due to the overestimated QTL effects that bias the real value of each QTL used in selection and finally to the fact that eBLUP based selection captures the final effect of all genes involved in trait expression, while MAS based on a few QTLs captures only a small portion of the variation. These results indicate that MAS for a few QTLs will hardly be useful as the sole selection criteria in forest breeding programs.
